# Lessons from the field: the conduct of randomized controlled trials in Botswana

**DOI:** 10.1186/s13063-017-2237-4

**Published:** 2017-10-27

**Authors:** Janice M. Bonsu, Rosemary Frasso, Allison E. Curry

**Affiliations:** 1 Master of Public Health Program, Perelman School of Medicine, University of Pennsylvania, Philadelphia, PA USA; 2 College of Population Health, Jefferson University, Philadelphia, PA USA; 3 Center for Research Injury and Prevention, Children’s Hospital of Philadelphia, Philadelphia, PA USA; 4 Department of Pediatrics, Perelman School of Medicine, University of Pennsylvania, Philadelphia, PA USA; 5 370 W. 9th Ave, Columbus, OH 43201 USA

## Abstract

**Background:**

The conduct of randomized controlled trials (RCTs) in low-resource settings may present unique financial, logistic, and process-related challenges. Middle-income countries that have comparable disease burdens to low-income countries, but greater availability of resources, may be conducive settings for RCTs. Indeed, the country of Botswana is experiencing a rapid increase in the conduct of RCTs. Our objective was to explore the experiences of individuals conducting RCTs in Botswana to gain an understanding of the challenges and adaptive strategies to their work.

**Methods:**

We conducted in-depth interviews with 14 national and international individuals working on RCTs in Botswana. Participants included principal investigators, research coordinators, lab technicians, research assistants, and other healthcare professionals. Interviews were audiotaped, transcribed verbatim, and coded for thematic analysis.

**Results:**

Five primary themes were identified: ethics board relationships (including delays in the process); research staff management (including staff attrition and career development); study recruitment and retention (including the use of reimbursements); resource availability (including challenges accessing laboratory equipment); and capacity-building (including issues of exporting locally sourced samples). These themes were explored to discuss key challenges and adaptive strategies.

**Conclusions:**

This study offers a first-hand account of individuals engaged in conducting RCTs in Botswana, a nation that is experiencing a rapid increase in research activities. Findings provide a foundational understanding for researchers in Botswana and trial managers in similar settings when planning RCTs so that the conduct of research does not outpace the ability to manage, support, and regulate it.

## Background

The randomized controlled trial (RCT) is critical to exploring the impact of interventions and is the ideal study design to establish causality [1]. Unique to the RCT design is the random assignment of participants to a treatment or intervention group, which reduces the potential for systematic biases that may compromise internal validity in observational epidemiologic studies [2]. RCTs have been critical to advancing public health and clinical research; many important medical treatments such as the well-known HIV drug, Tenofovir, have only been approved for population-wide use after a long series of RCTs [3].

The advantages of the RCT design have led to its widespread use. However, the conduct of RCTs in regions of the world with limited research capacity may present financial, logistic, and process-related challenges. For example, investigators in Lebanon faced barriers in enrollment that stemmed from misconception about unfamiliar terms such as “randomization” [4]. In Peru, researchers found that community members were hesitant to sign study consent forms due to cultural resistance to foreign documents as a result of past instances of land being unjustly seized by foreigners [5]. These few examples tell a greater narrative of cultural and linguistic challenges associated with the conduct of research in global settings. Studies have examined the utility of dedicated trial managers, individuals tasked with managerial and scientific duties, as critical to the success of trials [6]. However, that role is not standard in all studies.

It is critical to identify the factors that promote the conduct of high-quality RCTs, especially in regions of the world currently experiencing an increase in research activities. Botswana – a country in southern Africa – has undergone this exponential growth, particularly in clinical trials. This increase in research capacity may be attributed to the fact that while Botswana is an upper middle-income country, its diseases of burden such as HIV/AIDS and tuberculosis, are similar to that of low-income countries [7, 8]. Indeed, numerous research partnerships have formed between Botswana institutions and U.S. institutions to address these diseases, such as the: Botswana–UPenn Partnership, Botswana–Harvard Partnership, and Gates Foundation’s African Comprehensive HIV/AIDS Partnership [9–11]. This convergence of international partnerships and the Botswana government’s investment to support research has created a unique opportunity to not only do clinical trials, but also to study the conduct of RCTs in Botswana. Previous studies have primarily focused on the need for improved quality management systems and resource constraints in the region [12, 13]. This study aims to provide a qualitative account from individuals who are involved in the day-to-day conduct of trials in Botswana. Results would provide key insights for researchers who are engaged in Botswana and might provide a foundational understanding for those in similar settings.

## Methods

This qualitative study was conducted between June and October 2016 and was approved by the University of Pennsylvania Institutional Review Board (IRB) and the Botswana Health Research Development Committee (HRDC).

### Participant selection and data collection

We employed a snowball sampling approach to recruit respondents. To do so, the research team identified a contact organization (Botswana–UPenn Partnership) who then connected the first author with potential participants, some of whom facilitated additional contacts. Individuals were eligible for inclusion if they were involved in the previous or current conduct of RCTs in the catchment area of Gaborone, Botswana. The only restriction on participation was on distance from Gaborone, as recruiting nationwide would have posed logistic difficulties for interviews. Potential participants were first contacted via email in June 2016, with an additional email invitation two weeks later. Individuals who failed to reply to the contact attempts were assumed to have declined to participate in the study. Respondents were interviewed either in person or by telephone by the first author (JMB) in a private space away from the individual’s immediate work area, in order to maximize the privacy of the respondent. All interviews were audiotaped, transcribed verbatim, and de-identified.

### Qualitative interview instrument

The interview instrument was a semi-structured guide consisting of open-ended questions. All interviews were conducted in English with English-speaking respondents; thus, no translator was necessary. The interview guide included questions on RCT experience in Botswana and key aspects of the research process such as the IRB approval and participant recruitment and follow-up. This guide was piloted through mock interviews with volunteers prior to engaging the intended participants. Interviews were estimated to take 1 h. As is customary in qualitative research, early interviews informed amendments to the guide through an iterative process to explore additional areas of interest. The study team met monthly throughout the data collection period to assess saturation and quality control.

### Analytic approach

The research team employed a directed content analysis approach through which codes were developed in two ways: a priori (informed by the interview guide) and through line-by-line reading of five representative transcripts [14]. Each code was given an explicit definition in the codebook to ensure coding accuracy. The first author (JMB) independently coded all transcripts and a coding audit of four of the 14 transcripts was completed by the senior author (RF) to assess coding accuracy; percent agreement was assessed (range 98–100%). The analysis of the transcripts was facilitated by NVivo, a qualitative software analysis program [15].

## Results

Fourteen key informants participated in this study and their characteristics are described in Table 1. Their roles on the trial team included: principal investigator (*n* = 6); research coordinator (*n* = 2); laboratory technician (*n* = 2); research assistant (*n* = 3); and other healthcare professional (*n* = 1). Common themes dominated after 14 interviews, suggesting data saturation. The respondents had varying number of research projects conducted, ranging from one trial to twelve.

**Table 1:** Characteristics of key informants

Respondents	n	Column %
Overall	14	
Sex		
Male	6	43
Female	8	57
Nationality		
USA	7	50
Botswana	5	36
Other	2	14
Role in research		
Principal investigators	6	43
Other healthcare professional	1	7
Research coordinators	2	14
Laboratory technicians	2	14
Research assistants	3	22
Trials conducted (n)		
1–3	10	72
4–7	2	14
8+	2	14

Analysis revealed the following thematic categories: ethics approval; research staff management; study recruitment and retention; and resource availability and capacity-building. Data were then explored to identify the challenges within these codes and the adaptive strategies used by respondents, when possible, to navigate these challenges. These findings are described below with selected quotations identified by indentation and italicized text.

### Ethics approval

Respondents explained that the first step in conducting a study in Botswana is seeking ethics approval from the country’s institutional review board, the Health Research Development Committee (HRDC). Though their role is important in regulating studies in the country, many respondents discussed that the voluntary nature of the HRDC, whose reviewers have primary clinical and academic responsibilities, has led to significant delays in processing applications. As a result, there are no defined timeline for approval processes in the way that the USA or UK has, with some respondents citing periods a range of 6–9 months for determinations.

Respondents shared that the opening of the University of Botswana’s (UB) IRB has eased some delays in protocol processing by the HRDC, as the HRDC automatically approves any protocol approved by UB. However, some respondents still feel that there needs to be more done to accommodate the large volume of protocols. One respondent shared that upon a visit to the HRDC, he found that the delays in processing were due to a misplacement of his submission, as all submissions are handled in paper. Another respondent suggested that the HRDC should consider instituting financial incentives for the services of the reviewers, to encourage protocol determinations in a timely manner.

*What they need to do is invest some money in reform of the HRDC to fix it. Some countries charge money per protocol, I’m not saying that helps, but… I think they ask a lot of people’s time, so it’s hard to get people to engage in it.* (Principal investigator)

Despite these issues, respondents spoke of the benefits that have come from the HRDC, such as a mandate that research conducted by foreign investigators must include local collaborators. Most respondents felt that these partnerships with local collaborators greatly enriched their studies. They reported that local collaborators bring valuable knowledge from the community, informing teams on methods to increase acceptability of studies in the community. However, other respondents believe the mandate put too many demands on the few available local investigators, preventing meaningful collaborations.

### Research staff management

After gaining approval from the HRDC, the next phase of conducting a RCT is assembling research staff. Respondents frequently described issues of staff retention as a barrier to the conduct of research. Respondents reported that, as a result of short contracts, usually 6–9 months in length, tensions about job security have come to the forefront for staff. While employers assumed the issue of staff retention was a result of the short contracts, staff attributed the turnover to the lack of career growth opportunities within some research partnerships.

*When people leave [the bosses] don’t know why. People are leaving because they don’t see career development. You’re kind of stagnant. You don’t really advance.* (Laboratory technician)

As high turnover of staff is costly to the study in terms of time and money, respondents shared some practices that are aiding in staff retention. One respondent acknowledged that some research partnerships offer bonuses, referred to as “gratuity,” for those who complete their contracts. This gratuity encourages staff who may be otherwise looking for longer contracts not to leave the study prematurely. Additionally, respondents discussed the career development initiatives some research partnerships use to retain staff and build capacity. Respondents described that these initiatives include professional training seminars and routes for staff to pursue advanced degrees.

### Study recruitment and retention

After receiving necessary approval and assembling staff and research resources, the success of a study rests on designing and implementing effective recruitment and retention strategies. Respondents generally suggested moving away from passive forms of recruitment, such as admissions forms that are not always updated. Instead, respondents advocated that a more active form of recruitment, such as hiring specific recruitment staff, alleviated the burden from hospital nurses and physicians and improved recruitment rates. Respondents also found that integrating technology into the study enrollment process aided recruitment. One respondent shared that since the introduction of tablet technology to her enrollment process, she has been able to increase the speed at which she enrolls participants and, as a result, she has been able to recruit three times as many patients as before. However, these changes to support recruitment could come with unintended consequences. One respondent shared the misconception among some colleagues that effective recruitment was associated to the termination of employment.

*The person who I was working with had a very bad belief about research. They had the belief that if you are working in research, if you recruit more you will reach the target quickly and then you will be jobless.* (Research coordinator)

The most commonly referenced strategy to study recruitment and retention that was recommended by respondents was the use of reimbursements to offset the personal cost incurred for study participants. Respondents felt that compensating study participants for their time was a sign of respect and removed barriers to participating, such as travel. Though compensation increases retention, some respondents believed that monetary incentives could be coercive to study participants. To navigate this tension, a respondent shared that she only reveals the availability of reimbursements after an individual has expressed interest in the study.

*Sometimes we use [compensation] as part of recruitment. It should be the last thing. After probing whether I’m interested or not, then at the end you can say… don’t worry about coming here, because I’m going to give you transport money.* (Other healthcare professional)

Respondents explained that for some community members, research was a means to supplement income. One respondent recalled that participants would show up outside of their scheduled follow-up appointments because they were short on money and knew they would be given a reimbursement. Another respondent talked about participants who were selling study drugs, unaware of their randomization group (placebo or drug) and jeopardizing the health of others as well as the study results.

*Participants were very tricky… [they] were selling the drugs. And what was paining me was that they were not aware if they were taking the drug or the placebo. So, they would be selling to HIV-positive people, they would be coming with many stories: I was in a taxi, my drugs are taken… or I put them in the house, maybe the relatives took it.* (Other healthcare professional)

### Resource availability and capacity-building

As Botswana continues to engage in rigorous research, resource availability and capacity-building remain high-level challenges to the conduct of RCTs. Resource availability, in particular, was highlighted by many respondents. Respondents recalled study setbacks due to poor infrastructure and challenges accessing specialized laboratory equipment. One adaptive strategy that respondents use to circumvent these issues has been the sharing and coordination of resources by different research partnerships. These resources include facilities, equipment, and staff, which they singled out as the largest cost of any study.

Another respondent asserted that foreign researchers working in low-resource settings should assume the responsibility to improve local research capacity. To do so, the respondent spoke against the practice of exporting locally collected study samples internationally for analysis. The respondent also suggested a policy for the HRDC that would require researchers to send local research staff abroad when samples are exported, so that they may learn more about the techniques being used.

*I don’t think it’s quite right to take Batswana samples out of Botswana. I have a fundamental issue with samples collected from one country and taken to another, without any justifiable reason except convenience.* (Laboratory technician)

## Discussion

This paper offers an important perspective on research activities in Botswana and adds to the growing literature useful for trial managers conducting RCTs in all settings. Though respondents in our study worked with standard operating procedures, they discussed challenges in the “unwritten” parts of the protocol, such as ethics approval, staff management, and protocol adherence. Prior studies investigating the conduct of RCTs have reported similar challenges.

Clinical trial managers have recognized the inefficiencies that lie in reinventing the trial management wheel and suggest producing standard trial management guidelines to reduce those challenges. These standard guidelines would include the utilization of a trial manager in designing the study, assuring data quality, and ensuring that recruitment is realistic, practical, and ongoing [6]. A 2007 analysis of 114 multicenter trials found that less than one-third of trials recruited their original target within the time originally specified [16]. Clinical trial unit directors in the UK reported that improvements to trial recruitment, including methods to minimize attrition, were their highest priority [17]. Respondents in our study also highlighted recruitment and retention as a priority, sharing helpful strategies such as community collaboration in designing study advertisements and the provision of reimbursements. The use of reimbursements, however, were variable among our respondents, with some presenting it at differing points in the recruitment process (beginning or end). This type of variability may not only affect recruitment rates, but may also contribute to a differential selection bias.

Other studies have found success in recruiting from diverse populations when they integrate knowledge from local collaborators such as an RCT of indigenous people in four countries (New Zealand, Australia, Canada, and the United States). The inclusion of local collaborators, integration of community engagement groups to inform key study decisions, and the incorporation of local staff were strategies that our respondents also found to facilitate recruitment and retention [18]. Other factors that have been associated with good trial recruitment include having a dedicated trial manager, being a drug trial, and trials that address clinically important questions at a timely point [16]. Collectively, these studies highlight the importance of the role of local trial managers who are able to work collaboratively with partners in foreign settings.

This paper also uncovered issues of non-adherence to treatment protocols that threaten to undermine the RCT design. A review of 100 RCT publications from the *BMJ*, *New England Journal of Medicine*, the *Journal of the American Medical Association*, and *The Lancet* found that 98% of the trials studied reported non-adherence to treatment protocols [19]. The review found that adherence to randomized intervention is poorly considered in the reporting and analysis of published RCTs and often simply labeled as modified intention to treat. While researchers are still struggling on methods to optimize adherence, it is likely that using trial managers to investigate the causal factors of non-adherence could aid in the challenge.

Along with issues of study recruitment and ethics approval, the concept of capacity-building has been on the forefront of those engaging in international research. In our study, respondents identified tensions from behaviors such as exporting locally derived samples and the lack of career development as hindrances of capacity-building. The required capacity-building measures are diverse. Some efforts to build capacity in African countries experiencing a progressive rise in clinical trials include the creation of data safety monitoring boards to assist local researchers [20, 21]. These boards contribute to capacity-building as they train nationals in data safety monitoring and research skills training for research staff. It is critical for future work to encourage and evaluate such measures to establish progresses and shortcomings in capacity-building. Overall, we are encouraged by the adaptive strategies that respondents described and believe it is imperative that the conduct of research not only contributes to the generation of knowledge, but also to the strengthening of research systems.

### Strengths and limitations

The primary strength of this study is its inclusion of individuals representing a variety of roles within the trial team, resulting in a comprehensive collection of perspectives. This led to the collection of many themes, each of which can be further explored through subsequent studies (e.g. a study that narrowly focuses on exporting locally derived samples). Nonetheless, our findings must be considered in light of some limitations. In terms of transferability, we interviewed a convenience sample of individuals working on RCTs near the capital of Botswana, Gaborone. Though we believe this study can provide foundational understanding for researchers dealing with universal issues such as staffing, ethics approval, and recruitment, different regions may yield additional challenges and, thus, may reveal new adaptive practices. A final limitation rests in the exploratory nature of the study, as the authors are not currently based in Botswana with extensive work in the region, but rather, partnered with colleagues of the Botswana–UPenn Partnership to conduct this study. That said, this partnership served as a strength for this study as we were able to navigate our own ethics approval with guidance from nationally based research staff. Additionally, as we stated before, this partnership aided in our recruitment by providing us a platform to communicate with other researchers and research partnerships in the area.

## Conclusion

RCTs are a research design that is widely used to critically evaluate clinical treatments and interventions. This study assesses the conduct of RCTs in Botswana and provides meaningful, front-line data that can be shared with those working and regulating research in the region. Trial managers may find insight from this context that may be applicable to and potentially improve in their own trials. It is recommended that investigators systematically chronicle these experiences in papers to directly inform the work of the larger research community. This practice would have tremendous value for individuals who are engaged in building capacity in research infrastructure in global settings so that the conduct of research does not outpace the ability to manage, support, and regulate it.

## Abbreviations

HRDC: Botswana Health Research Development Committee
IRB: Institutional Review Board
RCT: Randomized controlled trial
UB: University of Botswana
